# Antibiotics in critically ill children—a narrative review on different aspects of a rational approach

**DOI:** 10.1038/s41390-021-01878-9

**Published:** 2021-12-06

**Authors:** Nora Bruns, Christian Dohna-Schwake

**Affiliations:** 1 Department of Pediatrics I, Neonatology, Pediatric Intensive Care Medicine, and Pediatric Neurology, University Hospital Essen, University of Duisburg-Essen, Essen, Germany; 2 Westdeutsches Zentrum für Infektiologie, University of Duisburg-Essen, Essen, Germany

## Abstract

**Abstract:**

Especially critically ill children are exposed to antibiotic overtreatment, mainly caused by the fear of missing out a severe bacterial infection. Potential adverse effects and selection of multi-drug resistant bacteria play minor roles in decision making. This narrative review first describes harm from antibiotics and second focuses on different aspects that could help to reduce antibiotic overtreatment without harming the patient: harm from antibiotic treatment, diagnostic approaches, role of biomarkers, timing of antibiotic therapy, empiric therapy, targeted therapy, and therapeutic drug monitoring. Wherever possible, we linked the described evidence to the current Surviving Sepsis Campaign guidelines. Antibiotic stewardship programs should help guiding antibiotic therapy for critically ill children.

**Impact:**

- Critically ill children can be harmed by inadequate or overuse of antibiotics.
- Hemodynamically unstable children with a suspicion of infection should be immediately treated with broad-spectrum antibiotics. In contrast, in hemodynamically stable children with sepsis and organ dysfunction, a time frame of 3 h for proper diagnostics may be adequate before starting antibiotics if necessary.
- Less and more targeted antibiotic treatment can be achieved via antibiotic stewardship programs.

## Background

Severe sepsis and septic shock remain a highly prevalent public health problem in critically ill children worldwide.^1^ In developed countries, the epidemiology of invasive bacterial infections, severe sepsis, and septic shock has changed over the last two decades.^2^ As a result of vaccination campaigns, invasive infections caused by pneumococci and meningococci have decreased but infections with gram-negative rods like *Klebsiella* have increased.^1,2^ Despite these successes in prevention, antibiotic therapy remains the main strategy to treat severe bacterial infections.

Additionally, the proportion of chronically ill children with bacterial infections requiring antibiotic treatment rose in recent years,^1,2^ leading to selection pressure and thus the development of an increased risk of selecting multi-drug resistant (MDR) bacteria. Bloodstream infections caused by MDR bacteria are associated with high mortality.^3^

Keeping the burden of severe bacterial infections in mind, the care for critically ill children presenting with systemic inflammation frequently includes antibacterial treatment, even if the viral infection is proven.^4^ A point prevalence investigation showed inappropriate antimicrobial therapy in 16–61% of cases on a cardiac and pediatric intensive care unit (PICU).^5^ The ARPEC point prevalence study including more than 1000 PICU patients worldwide revealed that 61% received at least one antibiotic (combination therapy in 51%).^6^ Prolonged (inappropriate) surgical prophylaxis was documented in 78% of patients.^6,7^ Overprescription of antibiotics can be driven, e.g., by the anxiety of missing an infection or not-challenging decisions of colleagues.^8^

As antibiotic overuse in PICU is frequent and adverse effects of antibiotics are well-known, a rational approach is needed. The Surviving Sepsis Campaign guidelines from 2020^9^ recommend fast initiation of antimicrobial therapy within the first hour of recognition in case of septic shock but leave 3 h of appropriate evaluation in children with sepsis-associated organ dysfunction without shock.

In this narrative review, we aim to summarize important aspects of antibiotic therapy in critically ill children that may help to reduce overtreatment. From these findings, we developed key questions for pediatric intensive care physicians that can support rational antibiotic decision-making in critically ill children. Wherever applicable, we put the existing evidence into context with the current Surviving Sepsis Campaign guidelines.

## Methodology

This review is based on an extensive search of scholarly/peer-reviewed literature. We conducted a PubMed search for literature published between 2000 and 2021. Articles were screened for relevance if they included an English abstract and the full text in either English, German or French. Search items included “critically ill children”, “PICU” and “antibiotic”, “therapeutic drug monitoring”, “biomarker”, “time to antibiotic”, “procalcitonin”, “sepsis”, “septic shock”, “surveillance”, “adverse events”. Relevant articles referenced in publications identified from our search were also reviewed. We included original research and reviews as well as expert opinions if they addressed one of the topics of our review.

## Results

**Fig. 1:**
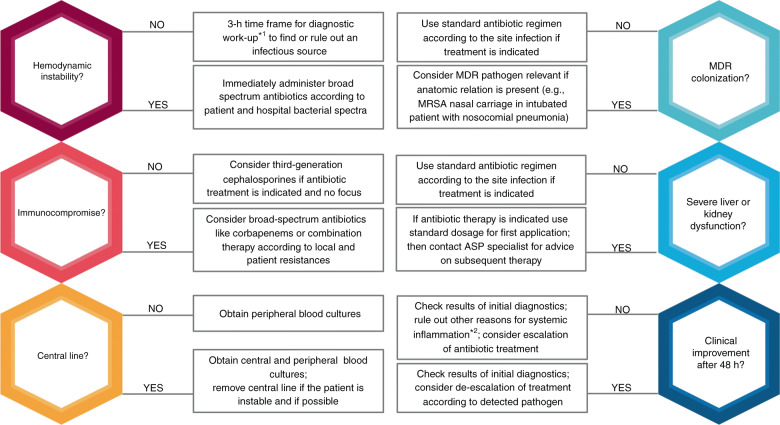
Key questions to guide decision-making in antibiotic treatment in critically ill children. MDR multi-drug resistant, ASP antibiotic stewardship program; *^1^ Septic workup: blood and urine studies including appropriate cultures, diagnostic imaging of the chest and abdomen/pelvis, if applicable: studies and appropriate cultures of tracheal fluid, cerebrospinal fluid and drains, wound swabs; *^2^E.g., viral infection, macrophage activation, pediatric inflammatory multisystem syndrome, etc.

Eighty-three original human studies were considered relevant for this narrative review. As evidence from the pediatric intensive care setting is limited, we included 19 adult studies. We structured the literature findings according to different topics (harm from antibiotics (*n* = 6/2), diagnostic approaches (*n* = 7/5), role of biomarkers (*n* = 6/3), timing of antibiotic therapy (*n* = 8/5), empiric therapy (*n* = 16), targeted therapy (*n* = 26/19), therapeutic drug monitoring (*n* = 14)), created a tabular overview over the studies (Supplementary Tables 1–7) and 6 key questions to aid in decision making on antibiotic therapy (Fig. 1).

## Harm from antibiotic treatment

This section will focus on harm from antibiotics in critically ill patient that extend beyond the selection of MDR bacteria, including the disruption of microbiomes, organ toxicity, leukocyte dysfunction, and idiosyncratic reactions.

### Disruption of the microbiome

Physiologic changes during critical illness and the use of antibiotics cause a loss of commensal colonization and an increase in potential pathogens in ICU patients, resulting in increased susceptibility to infection.^10–13^ These microbiome changes, known as dysbiosis, can directly cause disease, e.g., diarrhea and pseudomembranous colitis by *Clostridium difficile,* or lead to colonization with MDR bacteria via an increase of antibiotic resistance genes in the microbiome.^14–17^ In the long term, dysbiosis can drive pro-inflammatory mechanisms predisposing for subsequent diseases, such as asthma and obesity.^18,19^

### Organ toxicity

Several antibiotics have organ toxic side effects such as nephro-, oto-, and hepatotoxicity. Serum levels of antibiotics that exert dose-dependent organ toxicity, like aminoglycosides, glycopeptides, and polymyxins, are commonly subject to therapeutic drug monitoring.^12^ Other classes of antibiotics exert organ-specific toxicity, e.g., on the central nervous system (beta-lactams, macrolides) or the bone marrow (cotrimoxazole, linezolid).^12,20^ In non-critically ill adult inpatients receiving antibiotics, an average of 20% experienced at least one ADE, with risk increasing according to the longer duration of treatment.^21^ Among pediatric outpatients, an estimated 70,000 emergency department visits due to antibiotic ADEs occur annually in the United States of America, accounting for 46% of emergency department visits for ADEs.^22^

### Immune cell dysfunction

Detrimental effects of antibiotics on the innate and adaptive immune reaction have long been acknowledged.^23,24^ In these mechanisms, the common phylogenetic origin of mitochondria and bacteria may play a role, as important effectors of the innate immune response to pathogen infection are located at the mitochondrial surface.^25^ Antibiotics that fit mitochondrial carriers have the potential to impair the activity of the electron transport chain in mitochondria, thereby possibly attenuating immune cell function.^26–28^ However, the importance of these potential modulations of the immune system remains uninvestigated in critically ill patient, calling for further research.^12^

### Idiosyncratic reactions

While dose-dependent ADEs to antibiotics can be anticipated, immune-mediated idiosyncratic reactions are unpredictable. These immune reactions include anaphylaxis, eosinophilic skin rash, toxic epidermal necrolysis, Stevens–Johnson syndrome, and drug rash with eosinophilia and systemic symptoms (DRESS) syndrome.^29,30^ In children, the majority of antibiotic ADE visits to emergency departments were due to allergic or idiosyncratic reactions in a retrospective study.^22^

## Diagnostic approaches to identify pathogens

The gold standard for diagnostics of severe bacterial infections remain blood cultures or cultures of body fluids acquired from the infected organ system. Definite results are available 24–72 h after preservation and aid in modifying or terminating antibiotic treatment but not in the initial decision. Due to difficulties to obtain sufficiently sized samples in small children, it is not always possible to receive meaningful results. To tackle this problem, molecular diagnostics have been considered as promising tools to enhance pathogen detection. In several studies, a multiplex-polymerase chain reaction in addition to routine blood cultures detected more relevant pathogens than blood cultures alone,^31–33^ even in neonates when using micro blood samples (100 µl).^34^ However, it remains unclear if the higher rate of detection leads to better outcomes or even less antibiotic usage. The risk of contamination, limited number of detectable pathogens, lack of resistance testing, and high costs have to be outweighed against the undetermined clinical benefit.

Next-generation sequencing (NGS) for the detection of cell-free DNA in blood was developed as another promising approach to molecular detection of pathogens. The clinical value of NGS has been studied with promising first results in adults^35,36^ and a small single-center trial in immunocompromised children.^37^ However, the interpretation of results might be even more challenging than in multiplex-PCRs. More trials are on the way to scrutinize if NGS can contribute to a rational antibiotic therapy. High costs and limited availability must also be addressed before NGS can become clinical routine.

## Role of biomarkers in guiding antibiotic therapy

Currently used biomarkers for detection and follow-up of bacterial infections in children are C-reactive protein (CrP), Procalcitonin (PCT), leukocyte count, and especially in neonatology interleukin 6 and 8 (IL-6, IL-8). Here we focus on the use of PCT to guide antibiotic therapy. Procalcitonin as a biomarker is characterized by an early first peak in the course of a bacterial infection and a close relation to the clinical course and resolution of infection.^38^ In high-quality studies in adults, the use of PCT-guided algorithms reduced both mortality and antibiotic therapies in critically ill patients.^39–41^

A randomized controlled trial on the value of PCT for treatment guidance in neonatal early-onset sepsis showed a reduction of antibiotic treatment duration (64 vs. 51 h).^42^ There was no difference in mortality, which was attributed to the fact that no sepsis-related deaths occurred during the study.^42^ The results are limited by the fact that PCT was not compared to other biomarkers like CrP. In late-onset sepsis, the use of CrP showed fair discriminative power in a recent meta-analysis.^43^

The results of a randomized prospective trial in PICU patients did not confirm the reduction of antibiotic treatment duration as previously described.^44^ One difference to other studies was the integration of Antibiotic Stewardship recommendations in both study arms (PCT and usual care). This might have led to shorter antibiotic courses per se (at mean 6 days). A recent review gives a comprehensive overview on the utility and the challenges of PCT measurement in children with sepsis.^45^ When PCT was used for detection of bacterial infections thresholds, of 0.5 or 1 ng/ml were often used. For the cessation of antibiotic therapy, a threshold of a reduction of at least 80% from peak PCT levels is recommended.^45^

The current SSC guidelines review the value of procalcitonin (but not CrP) in the context of antimicrobial therapy duration. A daily clinical assessment including laboratory analyses for the possibility of antibiotic de-escalation is recommended. The authors conclude that PCT shows promise, but there is still insufficient evidence for algorithm-based treatment decisions.^9^

## Timing of antibiotic therapy

In patients with suspected systemic infection and arterial hypotension or life-threatening organ dysfunction, immediate initiation of antibiotic treatment is undisputed. In adults, some studies report linear increases of mortality with every hour of treatment delay, other studies report increased mortality after a certain delay, mostly between one and six hours.^46–48^ In children there is less evidence on the tolerable delay of treatment initiation. In one study conducted on a PICU, the critical threshold for a significantly increased mortality was three hours (adjusted odds ratio (OR) 4.8 (95% confidence interval (CI) 1.5–16.2)).^49^ Children with neutropenia after chemotherapy are especially prone to bacterial infections. In this population, administration of antibiotics within the first hour of check-in to hospital as a result of improved in-hospital processes significantly reduced the need for ICU consultation or admission.^50^

These studies highlight the importance of fast initiation of causal treatment in children with neutropenic fever, septic shock, or sepsis with organ dysfunction. Accordingly, the Surviving Sepsis Campaign calls for the initiation of empiric broad-spectrum antibiotic treatment within the first hour in septic shock and within three hours in sepsis-associated organ dysfunction.^9^

If completing the diagnostic work-up combined with watchful waiting may be justified in less critically ill patients remains yet to be answered: While some retrospective studies on bacteriemia in children failed to identify inadequate empirical antibiotic treatment as an independent risk factor for adverse outcome,^9,51,52^ another study found 2.9-fold (CI 1.2–7.0) increased adjusted odds for 30-day mortality if the empirical treatment was discordant.^53^ A retrospective cohort study of over 10,000 preterm infants found that non-indicated antibiotic treatment was associated with increased mortality.^54^ In a quasi-experimental pre-post design study, 101 adults with intensive care unit acquired infections treated on an intensive care unit immediately received antibiotic treatment during the first period of the study.^55^ During the second period, diagnostic work-up was completed and antibiotic treatment was only initiated after obtaining positive culture results in 100 patients. During first the period, days with antibiotic treatment (17.7 vs. 12.5 days) and adjusted odds for mortality (OR 2.5 (95% CI 1.4–4.0)) were significantly higher.

Some of these findings might support a watchful waiting strategy if no signs of septic shock or organ dysfunction are present. However, further evidence in this area is needed before a recommendation can be made.

## Empiric therapy

Broad-spectrum therapy is recommended in pediatric septic shock or sepsis-associated organ dysfunction to increase the likelihood that initial empirical treatment is effective. The number one choice in this context is broad-spectrum beta-lactams. However, choosing the adequate empirical antibiotic treatment becomes increasingly difficult if the prevalence of MDR bacteria is high, for example in hospital-acquired infections. To broaden the antimicrobial spectrum, a beta-lactam can be combined with an aminoglycoside if a gram-negative MDR infection is suspected, or with a glycopeptide, if gram-positive MDR bacteria are likely.

Treating all patients at risk for MDR infections sufficiently without exposing low-risk patients to unneeded organ toxicity drugs remains a challenge. For example, one retrospective study found a significant association of acute kidney injury with concomitant piperacillin/tazobactam and vancomycin treatment in children with suspected nosocomial infection.^56^ In another retrospective study, odds for acute kidney injury were doubled in children with gram-negative bacteremia receiving combination therapy with a beta-lactam and aminoglycoside compared to beta-lactam monotherapy.^57^ Mortality after 30 days was not affected.^57^ Even in severely ill (pediatric risk of mortality ≥ 15) or profoundly neutropenic (≤100 cells/ml) patients with gram-negative bacteremia, no 10-days survival benefit was reported for empirical combination therapy with a beta-lactam and an aminoglycoside.^58^ However, patients with MDR gram-negative bacteremia had reduced 10-days mortality (odds ratio 0.70, 95% confidence interval 0.51–0.84) and would benefit from empirical combination therapy.^58^ This study shows that the identification of patients at risk for MDR is extremely important to avoid the adverse effects of combination therapy and achieve the best outcomes at the same time.

Screening for carriage of multi-drug resistant bacteria has become routine in many health care facilities who care for critically ill child. Data on the association of positive swabs with subsequent invasive infection with the same pathogen are anecdotal to our knowledge.^59^ Nevertheless, some studies with mainly adult ICU patients assessed the relationship between preceding colonization and the probability of an infection. In general, critically ill patients with MDR carriage (mainly extended-spectrum β-lactamase pathogens) are at a clinically important risk for an invasive infection with the same bacteria.^60–63^ As a consequence, knowledge of colonization status could help improve the rate of adequate empiric antibiotic therapy. Whether this quality improvement leads to a better outcome in terms of survival, remains uncertain. Two of the four studies reported similar mortality rates but the number of patients was rather low, and mortality was not the primary outcome.^61,62^ The value of routine screening programs might also depend on the local incidence of MDR pathogens. A study by Jalalzai et al. in a low-endemicity ICU showed that it was safe to withdraw the routine screening program without harm.^64^ Additionally, the use of carbapenems was reduced during the period without screening.

The Surviving Sepsis Campaign recommends monotherapy with a third-generation cephalosporin for previously healthy children with community-acquired sepsis as initial empirical treatment.^9^ However, even in these patients, combination therapy with a glycopeptide is recommended in settings with a high prevalence of methicillin-resistant *Staphylococcus aureus* or ceftriaxone-resistant pneumococci. If ceftriaxone resistance is common in gram-negative bacteria, the addition of an aminoglycoside or substitution with a carbapenem is considered appropriate.^9^ In hospital-acquired sepsis or immunocompromised patients, the clinical history including comorbidities, recent antibiotic exposure, known colonization, and indwelling devices have to be considered for antibiotic choice. Initial antimicrobial treatment should consist of a broad-spectrum therapy with a single- or multi-drug regimen.^9^ If the patient is considered at risk for an MDR infection the above-described combination therapy should be applied.^9^

## Targeted therapy

Antibiotics are initiated because of a suspected infection. After obtaining results from cultures, an important strategy to avoid the development of MDR strains and reduce individual organ toxicity is a de-escalation of the initial empiric treatment. This includes targeted antimicrobial therapy whenever possible, discontinuation of treatment if no infection is confirmed, definition of treatment duration, and source control.

In a prospective multicenter study, children with neutropenic fever were stratified into groups with high, intermediate, and low risk for bacterial infection based on clinical findings and laboratory results. Patients in the high-risk group were treated empirically with antibiotics. Interleukin-8 was determined to distinguish between intermediate and low risk. Intermediate-risk patients received antibiotics and were re-evaluated after 72 h. In 41% of these patients, antibiotic treatment was discontinued following re-evaluation—without a case of sepsis relapse. Low-risk patients were clinically observed and discharged after 12 h without fever. However, 13% of the low-risk patients turned out to have an infection with coagulase-negative staphylococci requiring antibiotic treatment.^65^ Highlighting the adverse effects of unindicated antibiotic exposure, the failure to de-escalate initial empiric antibiotic treatment was associated with higher 90-days mortality in adults with severe sepsis or septic shock.^66^

A possible way to reduce antimicrobial exposure is the regular reassessment of indication for antibiotic treatment. In a tertiary pediatric intensive care unit, an antimicrobial time-out 48–72 h after treatment initiation led to defined treatment duration (63%) or discontinuation of treatment (29%) in most cases. Additionally, the time-out significantly reduced days of therapy per 1000 patient-days for vancomycin and meropenem compared to the pre-implementation period and reduced total acquisition cost for piperacillin/tazobactam, vancomycin, and meropenem.^67^ Similar findings have been reported on antimicrobial time-outs in non-critically ill children.^68^

The duration of treatment should be determined with respect to the site of infection, causative pathogen, response to treatment, and the possibility to control the source by surgical interventions or removal of infected catheters.^9^ It is important to point out that infections causing more severe initial illness or organ dysfunction do not generally require longer courses of treatment. In numerous studies, similar clinical outcomes were achieved after short courses compared to long courses of antibiotics.^69–77^ Long exposure to antibiotics can cause secondary complications such as necrotizing enterocolitis in very preterm infants, candidemia, infections with *C. difficile*, and development of antibiotic resistance, making unnecessarily long exposure to antibiotics obsolete.^78–82^

Besides the administration of antimicrobials, source control has been shown to be an important part of sepsis treatment also in children and should be intended whenever possible.^9,83–85^

These studies show that antibiotic treatment should be administered only as long as indicated, tailored to the patient’s needs, and de-escalated whenever possible. According to the Surviving Sepsis Campaign, assessment for de-escalation of antimicrobial therapy should be performed daily.^9^

## Therapeutic drug monitoring

If a decision in favor of antibiotic treatment has been made—be it adequate or not—an appropriate dosage should be administered to ensure an effective treatment. The adequacy of antimicrobial drug exposure is mainly determined by the volume of distribution and clearance. Sepsis patients are at risk for altered pharmacokinetics and pharmacodynamics due to shifts in distribution volume caused by fluid resuscitation, capillary leak, augmented or reduced renal clearance, and low serum albumin.^86–89^ These conditions typically occur during the initial phase of critical illness and resolve over time, thereby interfering with rigid dosing regimens that do not account for intraindividual pharmacokinetic variability.^90^

Several reports highlight the role of augmented renal clearance for subtherapeutic drug exposure to antibiotics with renal elimination profiles, such as glycopeptides, aminoglycosides, and beta-lactams.^90–94^ While therapeutic drug monitoring is well-established in clinical practice for vancomycin and aminoglycosides, evidence is increasing that there may also be a role for monitoring beta-lactam plasma concentrations. A retrospective study in critically ill children found that augmented renal clearance, early phase of sepsis, and severity of sepsis-associated illness were associated with subtherapeutic levels of beta-lactams.^86^ In this study, no case of concentrations above the threshold or clinical toxicity was observed and children receiving continuous infusion had adequate plasma concentrations.^86^ Further studies have linked augmented renal clearance and critical illness in children to subtherapeutic treatment exposure to beta-lactams.^95–102^

Therapeutic drug monitoring of beta-lactams not only helps to avoid organ toxicity but may also be a useful tool to assure adequate plasma concentrations of antimicrobial agents. Given the rapid changes in pharmacokinetics and pharmacodynamics children undergo during critical illness and subsequent recovery, individualized dosing strategies and monitoring of plasma concentrations of antimicrobial agents including beta-lactams seem inevitable in pediatric intensive care patients. Even though therapeutic drug monitoring will not reduce antibiotic overuse, it can help to reduce organ toxicity and ensure that the conducted treatments are effective.

## Conclusion

Antibiotics can save lives, but at the same time exert harm to the individual by multiple mechanisms, ranging from direct impairment of cellular function to disturbance of physiological homeostasis and even contributing to the development of new diseases. Awareness of these potential harms must become an essential part of physician’s decision-making for or against antibiotic treatment.

Critically ill children may be a patient group that needs wise decision-making on antibiotic therapies even more than others. Important aspects to consider on the path to adequate antibiotic treatment are diagnostic approaches to identify pathogens and guide antibiotic treatment, timing of antibiotics, empiric and targeted therapy, and therapeutic drug monitoring. To guide decision-making, we developed 6 key questions to answer before the initiation of antibiotic therapy in critically ill children.

## Supplementary information

Supplementary Table 1

Supplementary Table 2

Supplementary Table 3

Supplementary Table 4

Supplementary Table 5

Supplementary Table 6

Supplementary Table 7
