# Supplementary material for: Antibiotics in critically ill children—a narrative review on different aspects of a rational approach
Source: Pediatr Res. 2021 Dec 6;91(2):440–6. doi: 10.1038/s41390-021-01878-9 (PMC8816725; doi:10.1038/s41390-021-01878-9)
Supplement: Supplementary file 1 — Supplementary Table 1 [file 41390_2021_1878_MOESM1_ESM.docx]

Table 1: Harm from antibiotics

|  | **Study** | **Study population** | **N; age** | **Study type** | **Primary exposure/ intervention** | **Main outcome** | **Main results** |
| --- | --- | --- | --- | --- | --- | --- | --- |
| **Children** | |  |  |  |  |  |  |
|  | Lovegrove  2019 | Pediatric outpatients | 6542; ≤ 19 years | Observational retrospective | Antibiotic treatment with ADE presented in ED | Epidemiology and antibiotics causing ADE | 41% of ADEs occurred in children ≤ 2 years, 86 % involved allergic reactions, Amoxicllin had highest rates of ED visits in children ≤ <ears, sulfamethoxazole-trimetoprim had highest rates in children aged 10-19 years |
|  | Patrick 2020 | Children | 2644 and 917 <1 year | population-based and prospective cohort analyses | Antibiotic prescription in first year of life | Asthma incidence (2644 children); composition of gut microbiota (917 children) | Asthma incidence increased by 24% with each 10% increase in antibiotic prescribing; gut microbiota at age 1 year was a significant mediator between outpatient antibiotic exposure in the first year of life and asthma diagnosis at age 5 years |
| **Adults** | |  |  |  |  |  |  |
|  | Cammarota 2015 | Adult patients | 39 (stopped early); >18 years | Randomised clinical trial | *Clostridiodes difficile* infection treated with FMT or vancomycin | resolution of diarrhoea | After interim analysis stopped; in patients with FMT diarrhea resolution in 90%; in patients with vancomycin in 26% |
|  | Tamma 2017 | Inpatients admitted to general medicine | 1488; | Retrospective cohort study | At least 24 hours of any parenteral or oral antibiotic therapy | Frequence of antibiotic-associated adverse drug events | 20% of patients experienced at least one antibiotic-associated adverse drug event |
|  | Yeh 2016 | Adult ICU patients | 32; 50 years | Prospective | microbiota of different body sites | Bacterial 16S amplicon PCR and sequencing | depletion of important commensal bacteria and enrichment with pathogens such as Enterococcus, Mycoplasma, and Staphylococcus |
|  | Zaborin 2014 | Adult ICU patients | 14 | Prospective |  | composition of cultured species from stool samples | emergence of ultra-low-diversity communities (1 to 4 bacterial taxa) in some patients |

ADE = advese drug event, ED = emergency department, FMT = Fecal microbiota transplantation, ICU = intensive care unit, VPA = valproic acid
