# Supplementary material for: Antibiotics in critically ill children—a narrative review on different aspects of a rational approach
Source: Pediatr Res. 2021 Dec 6;91(2):440–6. doi: 10.1038/s41390-021-01878-9 (PMC8816725; doi:10.1038/s41390-021-01878-9)
Supplement: Supplementary file 2 — Supplementary Table 2 [file 41390_2021_1878_MOESM2_ESM.docx]

Table 2: Diagnostic approaches to identify pathogens

|  | **Study** | **Study population** | **N; age** | **Study type** | **Primary exposure/ intervention** | **Main outcome** | **Main results** |
| --- | --- | --- | --- | --- | --- | --- | --- |
| **Children** | |  |  |  |  |  |  |
|  | Gies  2016 | Children with SIRS under antibiotic treatment | 39; 5 years | prospective | Comparison of multiplex PCR with blood cultures | Rate of positive results | 14/39 positive multiplex PCR results compared to 4/39 positive blood culture results; adaption of antimicrobial treatment in 7/14 positive patients |
|  | Horiba 2018 | Immuno-compromised children with confirmed or suspected BSI | 35; age not given | Not stated | Next Generation Sequencing as diagnostic tool for BSI | Rate of positive NGS results in culture positive and culture negative BSI | Bacteria isolated in blood culture were identical to the dominant bacteria by NGS in 8 of 12 patients; NGS detected three pathogen in suspected BSI |
|  | Lucignano 2011 | Children in hospital with suspected sepsis | 803 children with 1673 samples; age not given | retrospective | Comparison of multiplex PCR with blood cultures | Rate of positive results | Positive results were higher with multiplex PCR (14.6%) than with blood cultures (10.3%); 97 additional isolates were identified by multiplex PCR |
|  | Tröger 2016 | Very low birth weight infants with suspected late onset sepsis | 133 with 214 samples; gestational age 25.6 weeks; age 19 days | Prospective multicenter | Comparison of multiplex PCR with blood cultures | Rate of positive results | PCR was positive in 110/214 episodes (51%) and blood culture was positive in 55 episodes (26%); Multiplex-PCR results influenced clinical decision making in 30% of episodes |
|  | Tschiedel 2012 | Critically ill children | 75 with 110 samples; 6 years | retrospective | Comparison of multiplex PCR with blood cultures | Rate of positive results | 26/110 positive multiplex PCR results compared to 19/110 positive blood culture results; in patients with antimicrobial treatment 24/110 positive PCR results vs 11/110 positive blood culture results |
| **Adults** | |  |  |  |  |  |  |
|  | Grumaz 2019 | Adults with septic shock | 50 with 256 samples; 66 years | Secondary analysis of prospective study | Comparison of Next Generation Sequencing of free microbial DNA with blood cultures | Rate of positive results | Positivity rate for NGS was 72%, for blood cultures 33%; NGS led to modification of antimicrobial therapy in 53% of patients |
|  | Grumaz 2020 | Adults with septic shock | 4 with 8 samples | Secondary analysis of prospective study | complete diagnostic workflow for real-time high-throughput sequencing of cell-free DNA from plasma based on nanopore sequencing for the detection of the causative agents | Establishment of workflow | 3.5-fold increase in sequencing throughput could be achieved; in all eight samples relevant pathogens were detected |

BSI = blood stream infection, DNA = deoxyribonucleic acid, NGS = next generation sequencing, PCR = polymerase chain reaction
