# Supplementary material for: Antibiotics in critically ill children—a narrative review on different aspects of a rational approach
Source: Pediatr Res. 2021 Dec 6;91(2):440–6. doi: 10.1038/s41390-021-01878-9 (PMC8816725; doi:10.1038/s41390-021-01878-9)
Supplement: Supplementary file 3 — Supplementary Table 3 [file 41390_2021_1878_MOESM3_ESM.docx]

Table 3: Role of biomarkers in guiding antibiotic therapy

|  | **Study** | **Study population** | **N; age** | **Study type** | **Primary exposure/ intervention** | **Main outcome** | **Main results** |
| --- | --- | --- | --- | --- | --- | --- | --- |
| **Children** | |  |  |  |  |  |  |
|  | Brown 2020 | Neonates with suspected late onset sepsis | 2255; mostly preterm babies, age not reported | Systematic review and meta-analysis | accuracy of serum CRP levels with microbiological culture results to diagnose late-onset sepsis | Sensitivity and specifity of initial CRP | At median  specificity (0.74), pooled sensitivity was 0.62; posttest probabilities of 26% for a negative test  result and 61% for a positive test result |
|  | Katz 2021 | Children on PICU with antibiotics | 270; 2.3 years in PCT arm vs. 1.6 years in usual care arm | Single center RCT | PCT guided antibiotic therapy vs. usual care | median  antibiotic days of therapy per patient in the first 14-days after enrollment | Antibiotic therapy days were 6.6 in the PCT arm vs. 7.6 in the usual care arm (n. s.) |
|  | Stocker 2017 | Neonates of gestational age 34 weeks or older, with suspected early-onset sepsis requiring  antibiotic treatment | 1710; gestational age 39 weeks | Multicenter RCT | procalcitonin-guided decision making or standard care-based  antibiotic treatment | non-inferiority for re-infection or death in the first month of life and superiority for duration of antibiotic therapy | Duration of antibiotic therapy in PCT group 55.1 hours vs. 65 hours in standard-of-care group. Non-inferiority for re-infection or death could not be shown due to the  low occurrence of re-infections and absence of study-related death. |
| **Adults** | |  |  |  |  |  |  |
|  | De Jong 2017 | Critically ill patients on ICU within 24 hours of antibiotic exposure | 1575; 65 years | Multicenter RCT | Discontinuation of antibiotics if PCT reduced by > 80% of peak or < 0.5 µg/L vs. standard-of-care | antibiotic daily defi ned doses and duration of  antibiotic treatment | Daily defined doses in PCT group 7.5 vs. 9.3 in standard-of-care group; mortality in PCT group was 20% vs. 25% in standard-of-care group |
|  | Schuetz 2017 | Patients with acute respiratory infections | 6708; 60.7 vs. 61.2 years; no pediatric study | Meta- analysis of RCTs | Antibiotic exposure based on PCT concentrations | 30-day mortality and setting-specific treatment  failure | Mortality was significantly lower in PCT-guided patients (9% vs. 10%) as was antibiotic exposure (5.7 vs. 8.1 days) |
|  | Wacker 2013 | Patients with SIRS, sepsis, severe sepsis and septic shock | 3244 including 177 children (>28 days) | Systematic review and meta-analysis | differentiation of septic patients  from those with a systemic infl ammatory response syndrome of noninfectious  origin | Sensitivity and specifity of PCT | Sensitivity was 0.77 and specifity was 0.79; sensitivity in pediatric studies ranged from 0.57 to 0.86, specifity ranged from 0.74 to 1 |

ICU = intensive care unit, PCT = procalcitonin, PICU = pediatric intensive care unit, RCT = randomized controlled trial, SIRS = systemic inflammatory response syndrome
