# Supplementary material for: Antibiotics in critically ill children—a narrative review on different aspects of a rational approach
Source: Pediatr Res. 2021 Dec 6;91(2):440–6. doi: 10.1038/s41390-021-01878-9 (PMC8816725; doi:10.1038/s41390-021-01878-9)
Supplement: Supplementary file 4 — Supplementary Table 4 [file 41390_2021_1878_MOESM4_ESM.docx]

Table 4: Timing of antibiotic therapy

|  | **Study** | **Study population** | **N; age** | **Study type** | **Primary exposure/ intervention** | **Main outcome** | **Main results** |
| --- | --- | --- | --- | --- | --- | --- | --- |
| **Children** | |  |  |  |  |  |  |
|  | Averbuch 2017 | Children with cancer | 80 patients with 107 episodes; 5 years | retrospective | Bacteremia with non‑fermentative gram‑negative rods | 30 day survival after first detection | Inappropriate antibiotic therapy in 23.4%; 30-day mortality 2.8% |
|  | Salstrom 2015 | Children with fever and neutropenia | 116; <18 years, mean or median not given | retrospective | Quality interventions to improve time to antibiotics | duration of fever, bacteremia, imaging  studies, ICU level care, LOS | Quality interventions achieved the goal of administration of antibiotics within the first hour which was associated with the need for ICU care |
|  | Ting 2016 | very low birth weight neonates | 11669; | retrospective | Antibiotic use without necrotizing enterocolitis or culture-proven sepsis | Mortality or major morbidity | A 10% increase in antibiotic usage rate was associated with an increased odds of the composite primary endpoint (OR 1.18) |
|  | Tsai 2014 | Neonates on neonatal ICU | 70 episodes of bacteremia; 31.5 days | retrospective | Bacteremia with MDR GNB bacteremia | Risk factors for MDR GNB and outcome | Independent risk factors for overall mortality were presence of infectious complications after bacteremia (OR: 3.16) and underlying secondary pulmonary hypertension with or without cor pulmonale (OR: 6.19) but not inappropriate antibiotic therapy |
|  | Weiss 2014 | Children with severe sepsis or septic shock | 130; 7.7 years | retrospective | Hourly delay from sepsis recognition | PICU mortality | Delay of >3 hours was associated with an odds of 4.84 for adjusted PICU mortality |
| **Adults** | |  |  |  |  |  |  |
|  | Joo  2014 | ED patients with severe sepsis or septic shock | 591; >18 years | Registry data | 3 hours from ED arrival | hospital mortality, 48-hour change in SOFA score, and LOS | Administration of antibiotics within first 3 hours was associated with an OR of 0.54 for adjusted in-hospital mortality |
|  | Liu 2017 | Inpatients at ED with sepsis | 35000; >18 years | retrospective | Administration of antibiotics within 6 hours of presentation | In-hospital mortality | Hourly delay of antibiotic administration leads to increased mortality in sepsis, severe sepsis and septic shock even within the first 6 hours |
|  | Peltan 2019 | ED patients with clinical sepsis | 10811; >18 years | retrospective | Door-to-antibiotic-time | 1-year mortality | Each additional hour of antibiotic delay was associated with a 10% increased odds of 1-year mortality |

ED = emergency department, GNB = gram-negative bacteria, ICU = intensive care unit, LOS = length of hospital stay, MDR = multidrug resistant, OR = odds ratio, PICU = pediatric intensive care unit, SOFA = Sequential Organ Failure Assessment
