# Supplementary material for: Antibiotics in critically ill children—a narrative review on different aspects of a rational approach
Source: Pediatr Res. 2021 Dec 6;91(2):440–6. doi: 10.1038/s41390-021-01878-9 (PMC8816725; doi:10.1038/s41390-021-01878-9)
Supplement: Supplementary file 5 — Supplementary Table 5 [file 41390_2021_1878_MOESM5_ESM.docx]

Table 5: Empiric therapy

| **Study** | **Study population** | **N; age** | **Study type** | **Primary exposure/ intervention** | **Main outcome** | **Main results** |
| --- | --- | --- | --- | --- | --- | --- |

| **Children** |  |  |  |  |  |
| --- | --- | --- | --- | --- | --- |

| Downes 2017 | children  hospitalized for 3 or more days | 1915;  with AKI: 12.6 years;  without AKI: 5 years | retrospective | IV therapy with vancomycin plus 1 other antipseudomonal  β-lactam combination therapy | Acute kidney injury on hospital days 3 to 7 within 2 days of receiving combination therapy | 8.2% had antibiotic-associated  AKI |
| --- | --- | --- | --- | --- | --- | --- |
| Flokas 2017 | Hospitalized children | 20234 | Systematic review and meta-analysis | VRE colonization and infection | Prevalence | Prevalence among hospitalized children and on PICU 5%, highest on hematology/oncology wards; OR 8.75 for developing subsequent VRE infection |
| Sick  2014 | Hospitalized pediatric patients with gram-negative bacteremia | 452; combination therapy 4 years, monotherapy 5.5 years | retrospective matched-pair analysis | empirical antibiotic combination therapy  versus empirical monotherapy | estimated odds of 10-day mortality and the relative  duration of bacteremia | No difference in mortality; no difference in duration of bacteremia; children with multi-drug resistant gram-negative bacteremia had OR of 0.7 of mortality |

| **Adults** |  |  |  |  |  |
| --- | --- | --- | --- | --- | --- |

| Baba 2011 | Adult ICU patients | 228 BSI | retrospective | nosocomial gram-negative BSI treated by piperacillin/tazobactam or meropenem | Colonization  concordance between MDR infection and previous colonization | Surveillance cultures predicted resistance in 52.9% and 51.4% of BSIs caused by resistant pathogens to  piperacillin/tazobactam and meropenem; no data on mortality |  |
| --- | --- | --- | --- | --- | --- | --- | --- |
| Blot 2005 | Adult ICU patients | 157 bacteremia episodes in 129 patients | retrospective | BSI caused by MDR bacteria | Infectious and microbiological characteristics and rates of appropriate antibiotic therapy in patients with and without colonization prior to bacteremia | 74.5% of bacteremia were preceded by colonization; appropriate antibiotic therapy (within 24 hours) in 75% of patients with known colonization vs. 55% without known colonization; appropriate therapy had no impact on outcome (44% vs. 38% mortality) |  |
| Cattaneo 2018 | Hematological cancer patients | 2226 including 144 with MDR (7 children) | prospective | MDR bacteria colonization and BSI | Prevalence | 37/144 patients (25.7%) colonized with MDR bacteria developed at least one BSI, MDR colonization has 6,5% prevalence and 16% prediction probability of BSI with the same pathogen |  |
| Jalalzai 2017 | Adult ICU patients | 524 during and 545 post active surveillance strategy | retrospective | Comparison of periods with active surveillance strategy for ESBL producing strains and without | incidence of ICU-acquired ESBL infections | No difference in ESBL infections; reduction in use of carbapenems |  |
| Papadomichelakis 2008 | Adult ICU patients | 34 VAP (31 MDR) and 104 BSI (55 MDR) | retrospective | Blood stream infections or ventilator associated pneumonia caused by MDR bacteria | Colonization  concordance between MDR infection and previous recent (<8 days) colonization | Concordance was 82% in VAP and 86% in BSI; Knowledge of previous colonization improved the rate of adequate empiric antimicrobial treatment (91 vs. 40%  in VAP and 86 vs. 50% in BSI cases); no difference of ICU mortality for BSI |  |

AKI = acute kidney injury, AUC = area under the curve, BSI = blood stream infection, ESBL = extended spectrum ß-lactamase, fT > MIC = percentage of 24 hours that the drug concentration exceeds the MIC, IV = intravenous, MIC = minimum inhibitory concentration, OR = odds ratio, PD = pharmacodynamic, PICU = pediatric intensive care unit, PK = pharmacokinetic, PTA = probability of target attainment, RIFLE = risk, injury, failure, loss of kidney function, end-stage kidney disease, q4 = fourth quartile, VAP = ventilator-associated pneumonia
