# Supplementary material for: Antibiotics in critically ill children—a narrative review on different aspects of a rational approach
Source: Pediatr Res. 2021 Dec 6;91(2):440–6. doi: 10.1038/s41390-021-01878-9 (PMC8816725; doi:10.1038/s41390-021-01878-9)
Supplement: Supplementary file 6 — Supplementary Table 6 [file 41390_2021_1878_MOESM6_ESM.docx]

Table 6: Targeted therapy

|  | **Study** | **Study population** | **N; age** | **Study type** | **Primary exposure/ intervention** | **Main outcome** | **Main results** |
| --- | --- | --- | --- | --- | --- | --- | --- |
| **Children** | |  |  |  |  |  |  |
|  | Adams 2019 | Pediatric patients on PICU | 95 patients pre-implementation, age 82 months, 95 patients post-implementation (age 78 months) | pre- and postimplementation quality improvement  study | Antimicrobial time-out after 48-72 hours | DOT per 1,000 patient-days for vancomycin, piperacillin/Tazobactam and meropenem | The cohort with time-out was exposed to less vancomycin (81.3 vs 138.1 days of therapy) and to less meropenem (34.7 vs 67.1 days of therapy) |
|  | Alexander 2011 | Neonates | 372; NEC: gestational age 28 weeks, age: 22 days | Retrospective case control study (1:2) | NEC | Role of antibiotic exposure | Exposure for >10 days resulted in a nearly three-fold increase in the risk of developing NEC  Antibiotic exposure without sepsis for >10 days resulted in a nearly three-fold increase in the risk of developing NEC |
|  | Bingöl-Kologlu 2007 | Children | 13; 35 months | retrospective | Necrotizing fasciitis | Diagnostic and therapeutic aspects | 12 patients survived and one patient with delayed diagnosis died |
|  | Celebi 2008 | Children on tertiary PICU | 102; died: 22 months; alive: 36 months | prospective | Nosocomial candidemia | risk factors, demographic features, treatment and  clinical outcome  risk factors, demographic features, treatment and  clinical outcome  risk factors, demographic features, treatment and clinical outcome | Independent risk factors  associated with candidaemia-related deaths by logistic regression analysis were  disseminated candidiasis (odds ratio, 5.7; P = 0.01), paediatric intensive care unit  stay (odds ratio, 8.1; P = 0.001), prolonged antibiotics therapy (odds ratio, 5.2;  P = 0.014), use of total parenteral nutrition (odds ratio, 4.4; P = 0.038) and  mechanical ventilation (odds ratio, 4.9; P = 0.01).  Independent risk factors associated with candidemia-related deaths were disseminated candidiasis (OR 5.7), PICU stay (OR 8.1), prolonged antibiotics therapy (OR5.2), use of total parenteral nutrition (OR 4.4) and mechanical ventilation (OR 4.9). |
|  | Chatterjee 2018 |  |  | Systematic review | Antibiotic resistance | Identification of risk factors | previous antibiotic exposure, underlying disease, and invasive procedures were the risk factors with most supporting evidence |
|  | Chowdhary 2006 | Neonates with blood-culture proven sepsis | 69; gestational age 35 weeks, age 12 days | RCT | Short (7 days) vs long (14 days) course of antibiotic treatment | treatment failure within 28 days  defined as positive blood culture, or clinical signs accompanied by either positive CRP or adjudicated  as relapse by an expert committee | trend to greater treatment  failures in the 7-day group compared with 14-day group (5 vs. 1); in *staphylococcus aureus* infection 4/7 treatment failures in short course vs 0/7 treatment failures in long course group |
|  | Dimopoulos 2008 | Adults and children with CAP | 4012 pediatric patient; 2-59 months | Meta-analysis of RCTs | Short (<8 days) vs long-course (2 or more days longer) antibiotic therapy | Clinical success defined as complete resolution or improvement or signs and symptoms of CAP | Clinical success for children did not differ between short and long course; OR 0.88 (n.s.) favors long course |
|  | Endorf 2012 | Children | 334; 7.9 years | retrospective | Necrotizing soft tissue infections | Characteristics, therapies and outcome | Longer time from admission to surgical debridement was an independent risk factor for mortality |
|  | Fustes-Morales 2002 | Children | 39; 4.4 years | Retrospective | Necrotizing fasciitis | Characterization of clinical features | Most frequent predisposing factor was malnutrition; Risk factor for mortality was immunosuppression |
|  | Hemels 2012 | Neonates with CONS sepsis | 142; gestational age of 30 weeks; sepsis onset at 9 (7-day treatment) vs 10 days (3-day treatment) | retrospective | Short course of vancomycin (3 days from 2008-2010) compared to long course (7 days from 2006-2008) for CONS sepsis | Recovery within 48 hours and CONS sepsis relapse | All infants recovered within 48 h and CONS sepsis did  not relapse |
|  | Karageorgopoulos 2009 | Children with bacterial meningitis | 426; 3 weeks to 16 years) | Meta-analysis of randomized trials | Short course (up to 7 days) vs long course (2 days longer or more than the current short course group) antibiotic treatment | complete recovery or substantial improvement of symptoms | No differences between groups; clinical success OR 1.24 (n.s.) favors short course |
|  | Lin 1985 | Children with bacterial meningitis | 79 | RCT | 7 vs 10 days of ceftriaxone treatment | Frequency of neurological complications | No differences between 7 vs 10 day treatment |
|  | Linder  2012 | Very low birth weight infants | 126; 15.8 days | retrospective | Vancomycin treatment for CONS | Sepsis recurrence after termination of antibiotic within 30 days | In total 2 CONS sepsis recurrence, none in the 5 day treatment arm |
|  | Martin 1990 | Children with bacterial meningitis | 119; 3 weeks to 15.5 years | RCT | Different duration of ceftriaxone course (short 4-7 days vs. full 8-12 days) | Complete clinical recovery | Complete clinical recovery was 91% in the short course group and 89% in the full course group |
|  | Miedema 2015 | Pediatric cancer patients with febrile neutropenia | 141 patients with 231 episodes | Prospective multicenter | Risk adapted approach for the need of antibiotic treatment | safety of the  risk assessment model in the low- and medium-risk  groups according to vital signs and interleukin-8 | No failure in the medium-risk group (shorten antibiotic treatment) but six failures in the low-risk group due to coagulase-negative *staphylococci* |
|  | Molyneux 2011 | Children with bacterial meninigitis | 1004 | RCT | 5-day vs. 10-day treatment course with ceftriaxone | Bacterial failure or relapse | 2 relapses in the 5-day group vs. 0 relapses in the 10-day group |
|  | Wirtz  2020 | Pediatric hospital | 177,984 antimicrobial orders in the pre-period and 173,377  in the  post-period | retrospective | mandatory antimicrobial indications and durations and a pharmacist-driven 48-  hour time-out | days of therapy per 1,000 patient-days | decrease in DOT per 1,000 patient days was observed for cefazolin (39.7 to 36.9 DOT), ampicillin (39.9 to 35.7 DOT), clindamycin (38.2 to 35.9 DOT), ceftriaxone (46.5 to 43.4 DOT), and meropenem (8.7 to 6.6 DOT) |
|  | Zaoutis 2010 | Children on PICU | 101; 3.4 years | population-based case-control study | Presence of candidemia | Risk factors for candidemia | Risk factors: presence of a central venous catheter (OR  30.4), malignancy (OR 4.0), use of vancomycin for >3 days in the prior  two weeks (OR 6.2), and receipt of agents with activity against anaerobic organisms for  >3 days in the prior two weeks (OR 3.5) |
| **Adults** | |  |  |  |  |  |  |
|  | Brown 2012 | Non-hospitalized patients | 2578; mainly adults | Meta-analysis of observational studies | Exposure to antibiotics | Impact on development of *Clostridium difficile* infection | OR for clindamycin: 16.8; for fluoroquinolones: 5.5; for cephalosporins, monobactams, and carbapenems: 5.68; macrolides: 2.68; sulfonamides and  Trimethoprim: 1.81; for penicillines: 2.71 |
|  | Garnacho-Montero 2014 | Adult patients with severe sepsis or septic shock | 628; 60 (alive) vs. 66 (dead) years | prospective | De-escalation of antibiotic therapy | In-hospital and 90-day mortality | De-escalation was associated with reduced odds (OR 0.58) of mortality at 90 days also if initial therapy was adequate (OR 0.54) |

CAP = community-acquired pneumonia, CONS = coagulase negative *staphylococci*, DOT = days of treatment, ICU = intensive care unit, MDR = multidrug resistant, NEC = necrotizing enterocolitis, OR = odds ratio, PICU = pediatric intensive care unit, RCT = randomized controlled trial, VRE = Vancomycin-resistant *enterococci*
