# Supplementary material for: Antibiotics in critically ill children—a narrative review on different aspects of a rational approach
Source: Pediatr Res. 2021 Dec 6;91(2):440–6. doi: 10.1038/s41390-021-01878-9 (PMC8816725; doi:10.1038/s41390-021-01878-9)
Supplement: Supplementary file 7 — Supplementary Table 7 [file 41390_2021_1878_MOESM7_ESM.docx]

Table 7: Therapeutic drug monitoring (only pediatric studies)

| **Study** | **Study population** | **N; age** | **Study type** | **Primary exposure/ intervention** | **Main outcome** | **Main results** |
| --- | --- | --- | --- | --- | --- | --- |
| Beranger 2018 | Children > 2.5 kg | 49; 23.7 months | prospective | Cefotaxime treatment | Cefotaxime pharmakonitetics; Monte Carlo simulations to maintain serum concentrations above the target concentration | Intermittent dosage reached target concentration in no patient; continuous infusion of 100 mg/kg/d reached a 100% probability of target attainment |
| Beranger 2019 | Children > 2.5 kg | 50; 2.3 years | prospective | Piperacillin treatment | Piperacillin pharmakokinetics; Monte Carlo simulations for targets: 50% fT> MIC and 100% fT> MIC | Only extended and continuous infusion reached predefined PK targets |
| Chosidow 2020 | Children with severe sepsis or septic shock | 37; 19 months | prospective | Treatment with ß-lactam antibiotics | ß-lactam plasma concentrations | 65% of patients had insufficient concentrations for a target of 100% fT > 4xMIC |
| Cies 2014 | Children on PICU | 13 with 31 samples; 9-months to 6 years | prospective | Different dosing regimens of Piperacillin/Tazobactam | probability of target  attainment (PTA) for piperacillin/tazobactam | Only dosing regimen to achieve 100% PTA for MIC of 16 mg/l was 100 mg/kg q6h as a 3-hour infusion and  400 mg/kg continuous infusion |
| Cies 2017 | Critically ill children | 9; 3.1 years | retrospective | Meropenem treatment | probability of target  attainment (PTA) for meropenem | Only 3- to 4-hour prolonged infusion and 24-hour continuous  infusion regimens were able to achieve PTA (40% fT > MIC) against all susceptible  Gram-negative bacteria |
| Cies  2018 | Critically ill children | 7 with 33 samples; 7 years | retrospective | Treatment for MRSA | Ceftaroline therapeutic drug monitoring | five of seven patients (71%)  demonstrated an increase in clearance, and 100% of patients  demonstrated a shorter half-life estimate as compared with the  package insert estimate; all patients showed a positive microbiological and clinical response |
| De Cock 2015 | Critically ill children | 50; 2.58 years | prospective | Amoxicillin/Clavulanic Acid treatment | Population pharmacokinetic analysis | 25 mg/kg (based on amoxicillin) q4 in children with cystatin C > 1 mg/l and q4 with infusion time of 1 hour in children with cystation C < 1 mg/l is best for PTA (40% fT > MIC 8 mg/l) |
| De Cock 2017 | Critically ill children | 47; 2.8 years | prospective | Piperacillin/Tazobactam treatment | probability of target  attainment (PTA) for piperacillin/tazobactam | 75 mg/kg (based on piperacillin) q4 h over 2 h, 100 mg/kg q4 h given over 1 h or loading dose of 75 mg/kg followed by continuous infusion of 300 mg/kg/24 h were minimal requirements for PTA (60% fT>MIC 16 mg/l) |
| Giachetto 2011 | Children on PICU | 22 | retrospective | Vancomycin treatment | Frequency of therapeutic range | 9/18 reached AUC/MIC > 400 on day 1 and 7/15 on day 3 |
| Gomez 2013 | Children with burns | 13 patients with 30 blood sample collections; 6 years | prospective | Vancomycin therapy | Vancomycin pharmacokinetics/ pharmakodynamics | The PK/PD target was reached for pathogens with 0.5 mg/L, 1 mg/L, 2 mg/L and 4 mg/L MIC in 93.3% (28/30), 66.7% (20/30), 33.3% (10/30) and 3.3% (1/30) of the sets, respectively |
| Hirai  2016 | Children with normal renal function | 109 | prospective | Vancomycin therapy | Presence of augmented renal clearance | Independent significant factors of vancomycin clearance were age and estimated glomerular filtration rate but not febrile neutropenia |
| Nichols 2016 | Children on PICU | 12 patients with 72 samples; 5 years | prospective | Extended infusions of Piperacillin/tazobactam treatment | 50% fT > MIC was calculated for different MICs | All extended-infusion regimens achieved probability of target attainment of > 90% at MICs of < 16 mg/l |
| Silva  2012 | Pediatric oncologic/hematologic intensive care patients | 31 patients with 61 measurements | retrospective | Vancomycin treatment | Vancomycin AUC/MIC > 400 (with MIC > 1 µg/ml) | AUC/MIC > 400 was obtained in 34 out of 61 dosages (55.7%), reached with a dosage of 81 mg/kg/d |
| Tamma 2013 | Hospitalized pediatric patients with gram-negative bacteremia | 879; combination therapy 5.7 years, monotherapy 6.4 years | retrospective | Antibiotic combination therapy | 30-day mortality and nephrotoxicity  classified according to the pediatric RIFLE | No association between combination therapy and mortality; patients receiving combination therapy had OR of 2.15 of nephrotoxicity |

AKI = acute kidney injury, AUC = area under the curve, BSI = blood stream infection, ESBL = extended spectrum ß-lactamase, fT > MIC = percentage of 24 hours that the drug concentration exceeds the MIC, IV = intravenous, MIC = minimum inhibitory concentration, OR = odds ratio, PD = pharmacodynamic, PICU = pediatric intensive care unit, PK = pharmacokinetic, PTA = probability of target attainment, RIFLE = risk, injury, failure, loss of kidney function, end-stage kidney disease, q4 = fourth quartile, VAP = ventilator-associated pneumonia
